# Detection of Explosives in a Dynamic Marine Environment Using a Moored TNT Immunosensor

**DOI:** 10.3390/s140304074

**Published:** 2014-02-27

**Authors:** Paul T. Charles, André A. Adams, Jeffrey R. Deschamps, Scott Veitch, Al Hanson, Anne W. Kusterbeck

**Affiliations:** 1 Center for Bio/Molecular Science and Engineering, Naval Research Laboratory, Washington, DC 20375, USA; E-Mails: andre.adams@nrl.navy.mil (A.A.A.); Jeffrey.deschamps@nrl.navy.mil (J.R.D.); anne.kusterbeck@nrl.navy.mil (A.W.K.); 2 Naval Undersea Warfare Center, 1176 Howell Street, Newport, RI 02841, USA; E-Mail: scott.veitch@subchem.com; 3 SubChem Systems, Inc., 65 Pier Road, Narragansett, RI 02882, USA; E-Mail: hanson@subchem.com

**Keywords:** 2,4,6-trinitrotoluene (TNT), explosives, immunosensor, seawater, fluorescence, remote

## Abstract

A field demonstration and longevity assessment for long-term monitoring of the explosive 2,4,6-trinitrotoluene (TNT) in a marine environment using an anti-TNT microfluidic immunosensor is described. The TNT immunosensor is comprised of a microfluidic device with 39 parallel microchannels (2.5 cm × 250 μm × 500 μm, L × W × D) fabricated in poly(methylmethacrylate) (PMMA), then chemically functionalized with antibodies possessing a high affinity for TNT. Synthesized fluorescence reporter complexes used in a displacement-based assay format were used for TNT identification. For field deployment the TNT immunosensor was configured onto a submersible moored steel frame along with frame controller, pumps and TNT plume generator and deployed pier side for intermittent plume sampling of TNT (1h increments). Under varying current and tidal conditions trace levels of TNT in natural seawater were detected over an extended period (>18 h). Overnight operation and data recording was monitored via a web interface.

## 1. Introduction

Long-term monitoring of estuaries, bays and rivers for the presence of toxic pollutants has for many decades been an important issue for continued sustainability of the marine ecological system. Toxic industrial chemicals (TICs), polychlorinated biphenyl's (PCBs) and heavy metals from industrial wastewater runoff, contaminated soils and groundwater plumes have the potential for creating an environment detrimental to marine, plant, and human kind. Of concern to the Department of Defense (DoD) and the Department of Navy (DoN) has been the potential impact on the marine environment of explosive substances from military exercises conducted in years past. Such exercises have left some shorelines and waterways cluttered with unexploded ordnance (UXO) leading to their designation as Superfund sites by the U.S. Environmental Protection Agency (USEPA) because of their high contamination levels. Documented reports and findings by the USEPA have revealed that contamination levels from explosives in these soils and groundwater far exceed USEPA thresholds [1–4]. Knowing these toxic levels exist has prompted agencies to ponder whether these explosive substances and other toxic compounds can leach into the marine environment and what possible risks (environmental and human) they might pose.

The primary component 2,4,6-trinitroluene (TNT), ubiquitous in munitions manufacturing, is well known for its potency as an explosive, but much less touted is the material's inherent toxicity and carcinogenic potential. With long-term exposure it is thought that such compounds could potentially impact coastal plant and marine life [5–7]. While the debate continues, evidence is mounting that supports the carcinogenic effects of TNT in humans, as well as its potential to affect the liver and spleen [8]. As environmental agencies seek to address the ecological impact that explosive substances may have on the marine environment, additional concerns have been brought to light relative to the safety and security compromises that the presence of explosive substances present.

Explosive substances found in the marine environment can signal compromised UXOs [8] that have the potential of exploding in the vicinity of ships or commercial watercrafts as they traverse harbors, ports, and thoroughfares. Although, it is extremely rare for an explosive event to occur as a result of a UXO displaced from the seafloor, this possibility amplifies the need for field-deployable long-term monitoring systems that can provide assurances to military personnel and civilians that their ports and harbors have safe passageways.

Traditional laboratory based analyses for explosives detection have shown improvements in specificity and sensitivity over the past decade [9–11]. However, many of the bench-top systems have the disadvantage of not being durable or field-deployable. Often such instruments require extensive personnel training to operate which is acquired only after many years of experience. Within the past few years scientist have adapted many explosives detection systems for field use. Ion-mobility spectrometers (IMS) [12], fluorescent polymers integrated into IMS systems [13–15], electrochemical sensors [16–18], and immunosensors [19–21] all have shown promise, but much work remains before they can be used long-term and perform at maximum efficiency in a marine environment.

Our research group has focused its efforts on the development of a microfluidic immunosensor for the detection of TNT that is sensitive, durable and field-deployable. The system also offers a number of other advantages: (1) high surface area-to-volume ratios for improved antibody-antigen interactions; (2) higher sampling flow rates (up to 9.0 mL/min); and (3) high specificity resulting from functionalized surfaces containing high affinity antibodies specific for TNT [20,21]. Using a fluorescence-based displacement format, the TNT immunosensor has effectively measured concentration levels for TNT in the parts-per-trillion (pptr) range with longevity that affords >100 analyses/device under operation that involves continuous seawater sampling [21]. Described herein are the field tests and results from employing the TNT immunosensor that has been engineered for underwater environmental assays to detect trace levels of TNT under naturally occurring tidal and current conditions. This field demonstration highlights the extraordinary binding capability and long-term field durability of the immunosensor in a complex matrix. Such an effort lends this sensing device as a potential platform for continuous environmental monitoring under real-world scenarios.

## 2. Experimental Section

Monoclonal antibodies (mAb; clone 30-1) specific for TNT were developed by Cell Essentials, Inc. (Boston, MA, USA). Tetraethylorthosilicate (TEOS), dimethylsulfoxide (DMSO), 3-mercaptopropyl-trimethoxysilane and TRIS buffers were purchased from Sigma-Aldrich/Fluka Corp (Milwaukee, WI, USA). Non-hazardous explosives for security and training and testing (NESTT) were obtained from XM (Rancho Cucamonga, CA, USA). 2,4,6-Trinitrotoluene (TNT) was purchased from Cerilliant, Inc. (Round Rock, TX, USA). 2,4,6-Trinitrobenzenesulfonic acid (TNBSA; 5% v/v) and *N*-succinimidyl-4-maleimidobutyrate (GMBS) were obtained from Pierce Chemical (Rockford, IL, USA). AlexaFluor647 cadaverine disodium salt was purchased from Invitrogen Molecular Probes (Carlsbad, CA, USA). Alexafluor-cadaverine-trinitrobenzene (AF-Cad-TNB) was synthesized and purified following previously published protocol [20]. Seawater was obtained from Narragansett Bay, RI, USA.

### 2.1. Assembly of Submersible Moored Steel-Frame Platform

**Figure 1.**
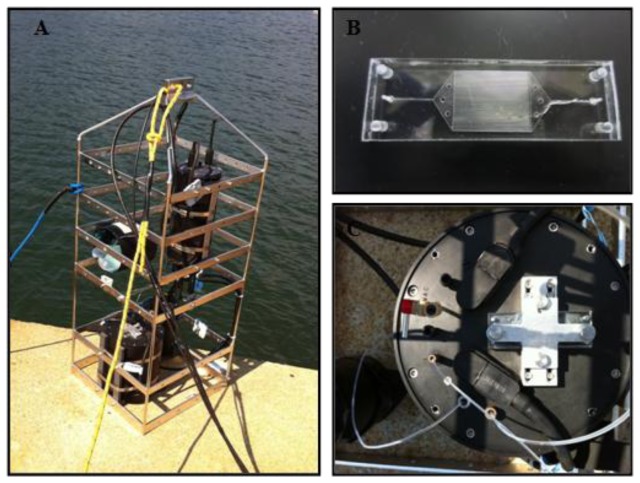
(**A**) Image of moored steel subframe with mounted microfluidic immunosensor. (**B**) Image of microfluidic device prepared in poly(methylmethacrylate) PMMA. The device contains 39 parallel microchannels (2.5 cm × 250 μm × 500 μm) functionalized with antibodies possessing a high affinity for TNT. (**C**) Top view of mounted TNT microfluidic device with inlet sampling and outlet ports for fluid passage to optical components.

The platform used to secure the microfluidic immunosensor device for underwater operation was a moored steel SubFrame15 (Figure 1A) constructed and engineered by SubChem Systems, Inc. (Narragansett, RI, USA). The subframe (38 cm × 38 cm × 106 cm) was a welded electro-polished unit specifically designed to accommodate a wide range of oceanographic instrumentation in conjunction with a SubChem Instrument Interface System (SIIS) which provide multiplexing bidirectional communication capabilities. The SIIS consisted of three parts, a submersible component, a tether, and a topside component. This design allowed it to integrate oceanographic devices with analog output and serial communication (RS232/485) to a single platform with communication packetized and transmitted to and from a topside component. The topside component was configured with GPS input capability for correlating position with data. In a moored application or mobile platform application, the GPS data can be easily used for tracking and navigating. The topside component of the SIIS was designed to enable use of several mediums of communication with the submerged component (SIIS.S1). The SIIS is also configured with a standard eight conductor tether. System power is provided and data were transmitted bi-directionally using RS485 at 115,200 baud. Communication with the topside component was accomplished locally using a serial port or internet through a secure IP web address. Web access was established either by RJ45 connection to a LAN, by satellite, or by CDMA cellular connection. The adapter collar provides access to the electrical, mechanical and optical sensor attachments. EcoPuck Triplet provided optical measurement capability for measurement of the AF-Cad-TNB dye conjugate.

### 2.2. Microfluidic Immunosensor Device Fabrication

The microfluidic device used to facilitate sensing was fabricated through a sequence of steps involving high precision micromilling and hot embossing [21]. In brief, a molding tool was milled from brass feedstock and used to hot emboss poly(methylmethacrylate) (PMMA). The devices had 39 microchannels (2.5 cm × 250 μm × 500 μm, L × W × D, Figure 1B). PMMA was the polymer of choice based on its inherent physical characteristics (durability, biofouling resistant, and amenable to immunomodification). PMMA was thermally annealed at 90 °C using a tri-solvent cocktail (47.5% DMSO, 47.5% DI H_2_O, and 5% methanol) creating the sealed microfluidic device. As a quality control measure, each device was leak tested then stored at room temperature until surface modification with antibodies specific for TNT was performed.

Antibodies specific for TNT were covalently immobilized to the interior walls of the microfluidic device using a protocol previously described [20]. This process involved coating the microchannels with a TEOS sol-gel solution forming a “glass-like” layer for silane-based functionalization. Use of a thiol-terminated silane (3-mercaptopropyltrimethoxysilane) enabled conventional covalent crosslinking chemistries for attachment of the heterobifunctional crosslinker, GMBS. The terminal NHS-ester of the GMBS crosslinker provided the final moiety for conjugating the antibody specific for TNT to the microchannel surface. Upon completion of the antibody immobilization step, a fluorescent analog of TNT (AF-Cad-TNB) was infused into the microchannels and allowed to saturate antibody binding sites. For long-term storage the microfluidic device was saturated with a 1% solution of bovine serum albumin (BSA) prior to dye saturation and stored at 4 °C. Prior to deployment the microfluidic device was mounted to the base module, face sealed to the payload and secured by hand tightening the spanner plate to the face (Figure 1C).

### 2.3. Immunosensor Calibration

Prior to deployment, calibration of the TNT microfluidic immunosensor was conducted using standards prepared from NESTT (1.0, 10, 25, 50 and 100 ppb) in buffered (0.01 M Na_3_PO_4_; pH 7.4; 0.1% Tween 20) natural seawater (performed in triplicate). Blanks consisted of buffered seawater containing no TNT. Optical parameters were preset for the AF-Cad-TNB dye conjugate with excitation and emission wavelengths at 632 nm and 665 nm, respectively. Fluorescent signal responses three standard deviations above background were deemed positive responses to TNT.

### 2.4. Field Deployment and TNT Sensing

**Figure 2.**
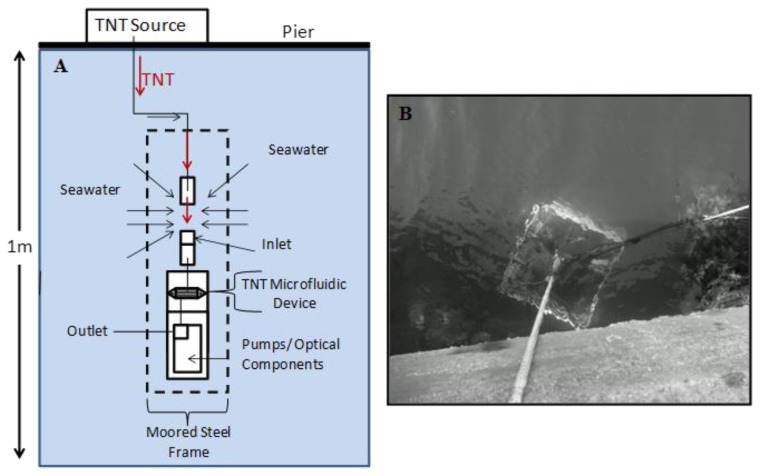
(**A**) Schematic of field demonstration configuration. TNT source container located on pier (topside) equipped with Teflon tubing and pump for supply of TNT near inlet of microfluidic device. TNT is pumped in and mixed with seawater before being drawn into inlet of device. Fluid delivery to flow cell for optical measurements was achieved with internal pumps while moored steel frame allowed a constant flow and mixing with natural seawater. (**B**) Pier-side deployment of TNT microfluidic immunosensor at 1 m depth below surface.

The anti-TNT modified microfluidic immunosensor was mounted to the payload and secured tightly to ensure no system leakage and proper fluid flow through inlet and outlet ports. The moored payload provided the instrumentation (sample pump, calibration/plume and buffer pump and optical components) necessary to control fluid flow through the microchannels, mixing of buffered solutions for optimum pH balance for antibody-antigen interactions, sample mixing of TNT in seawater and fluorescence measurements. TNT plume generation was achieved by pumping a solution of TNT (1 ppm) through an attached tethered line of Teflon tubing from a previously prepared TNT stock source (extracted from NESTT) kept topside on the pier. An intake port to the sensor was configured in close proximity to the outlet port of the pumped TNT source line to allow mixing with the natural seawater environment (schematic-Figure 2A). The submersible steel frame mounted with the TNT microfluidic immunosensor was deployed pier side at a depth of 1 m (from the top of the steel frame to the water surface) (Figure 2B) and tethered using two point mooring to prevent the frame from spinning. The frame was oriented such that the immunosensor sample intake was located downstream of the plume outlet in a flooding tide. The plume generation was preprogrammed for intermittent releases of TNT (10 mL; 1 h increments) into seawater. The plume pump was calibrated and set at a flow rate of 10 mL/min for 1 min to provide a sufficient dose of TNT. Incremental TNT sampling was programmed for 3 min time frames. To establish an initial baseline, analysis was also performed for 3 min where TNT was not pumped from the source. The flow rate and concentration of TNT was held constant throughout the field demonstration to measure sensor response through current and tidal cycles.

## 3. Results and Discussion

**Figure 3.**
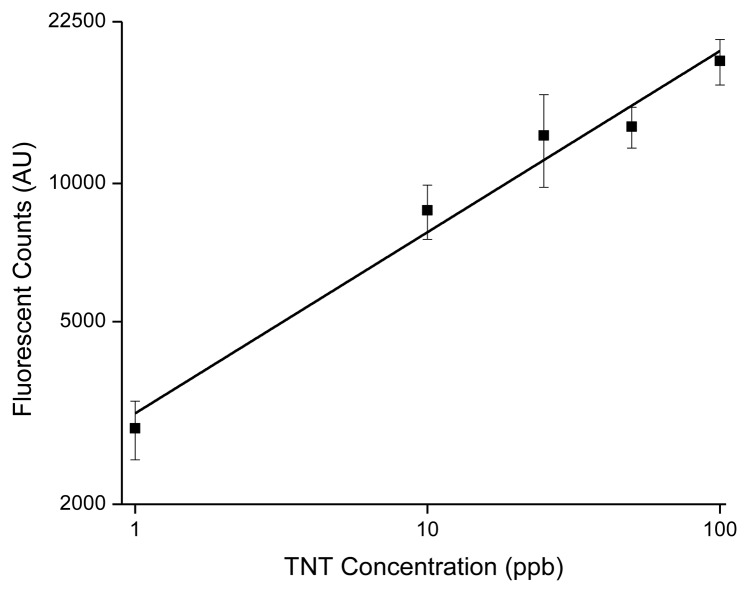
Calibration curve for TNT in natural seawater with the microfluidic immunosensor.

Figure 3 shows the calibration plot for the TNT concentrations applied to the microfluidic immunosensor.

**Figure 4.**
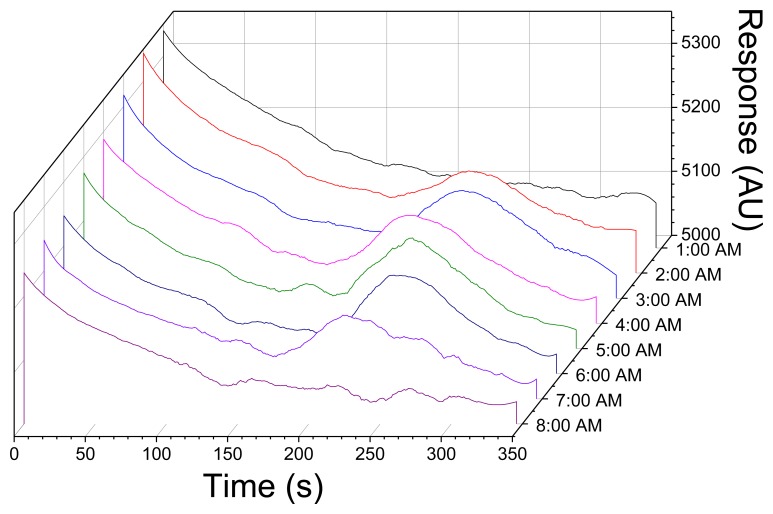
Waterfall chart showing fluorescence response to TNT from microfluidic immunosensor during an ebbing tide with corresponding tidal data. Analysis shown for TNT responses between 1:00 am–8:00 am (slack tide occurred at 1:18 am and 6:54 am).

The TNT microfluidic immunosensor provided a linear dynamic range that extends over two orders of magnitude with a calculated RSD of 12% (R^2^ = 0.97). Once submerged, tethered, and oriented at a depth of 1 m, the microfluidic immunosensor was operated overnight (approximately 18 h) to detect trace levels of TNT. The waterfall chart shown in Figure 4 is a compilation of positive immunosensor responses to TNT measured in real-time at intermittent sampling points through the tidal cycle from 1:00 am to 8:00 am (where slack tide occurred at 1:18 am and 6:54 am). Initial deployment of the immunosensor showed no signal response for TNT as the tide was flooding (9:00 pm–1:17 am).

**Figure 5.**
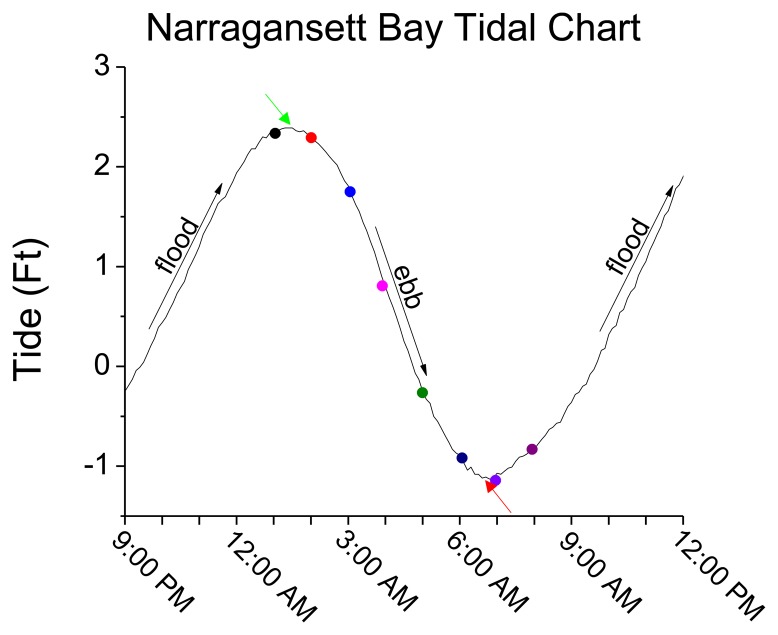
Semi-diurnal tidal data with color and time coordinated markers corresponding to Figure 4 shown with tidal direction and level. (Slack tide is marked by red and green arrows; ebbing tide is marked by black arrow with negative slope; and flooding tide is marked by black arrows with positive slope).

As shown in Figure 5, tidal changes occurred during the field demonstration (flood-slack-ebb-slack-flood), resulting in changes in directional flow over the course of the experiment. During a flooding tide the released TNT was carried away from the sipper before it could be sampled and quantified. Positive TNT responses were recorded consecutively by the immunosensor from 2:00 am–8:00 am where a slack tide or an ebbing tide was present.

It is believed that the TNT was carried to the inlet by the current or had time to diffuse to the inlet during ebbing or slack tides. The maximum amplitude for TNT detection occurred at 5:00 am. Each response shown corresponds to a plume simulation of 1–100 ppb TNT emanating from the plume generator where the released TNT was 1.0 ppm. Previous field experiments where the immunosensor payload was integrated with a REMUS AUV to transport and assess TNT composition in seawater suggested that the dilution effect of the seawater with a 2.54 cmseparation between the immunosensor inlet and plume release port could be as high as 100-fold [22].

**Figure 6.**
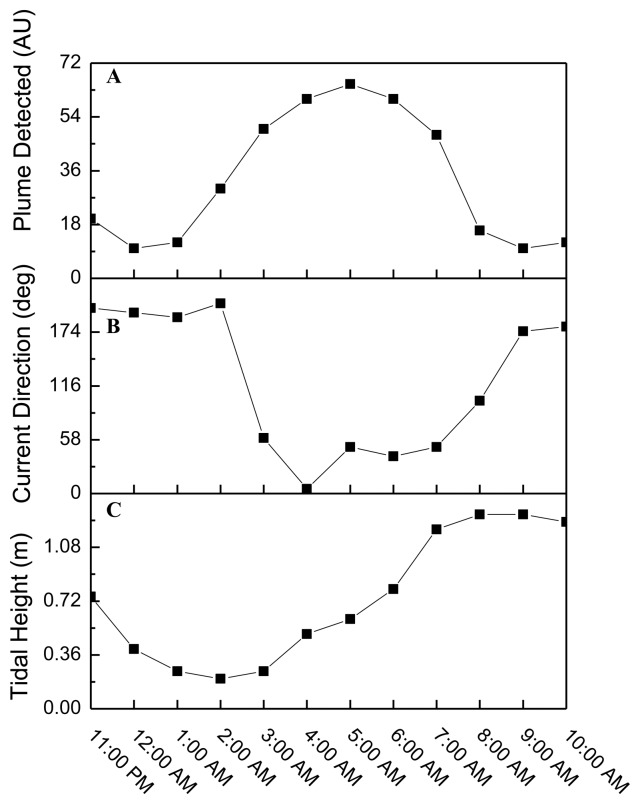
Plots illustrating the sensor response, current direction and tidal height are shown. (**a**) The time correlated microfluidic immunosensor response to TNT is shown over a 12 h period (11:00 pm–10:00 am); (**b**) current directional changes; (**c**) tidal height changes.

Figure 6 shows the sensor response, current direction, and tidal height. Given the relationship between the plume source and the immunosensor as fixed suggests the sensors response was intimately coupled to the current direction. More specifically, Figure 6B shows the current direction changed with the tide (Figure 6C). As the tide goes from slack to ebb the current direction was initially ∼197.5 (11:00 pm–2:00 am) then decreased to ∼41 (3:00 am–7:00 am). As the tide flooded near the end of the field demonstration the direction was ∼152. The sensor path was collinear with ∼41 line at 5:00 am as indicated by the observation of the highest signal corresponding to that time. Based on the sensor response (Figure 6A) the current direction flowed from the plume source towards the sensor during the flood-ebb cycles (2:00 am–8:00 am).

**Figure 7.**
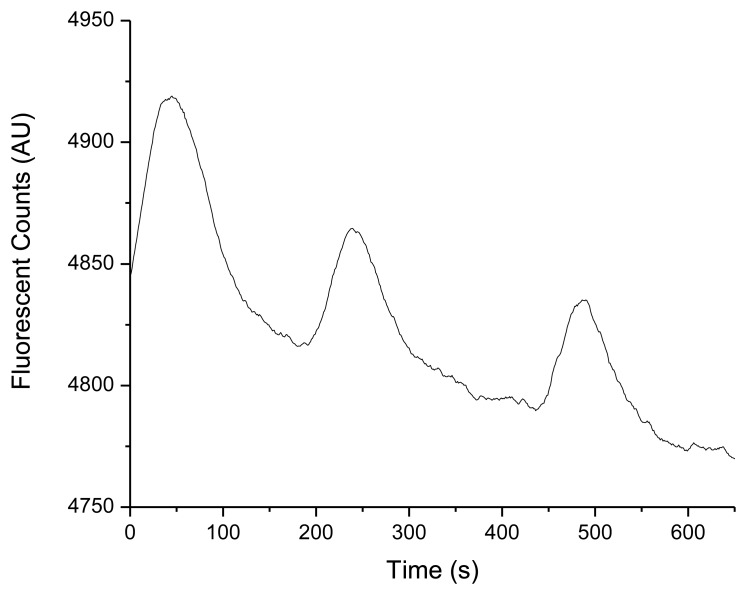
Fluorescence signal responses from immunosensor from triplicate injections of TNT (100 ppb) in natural seawater (post-deployment). The measured fluorescence response indicated the fluorescence dye reporter complex was not depleted from the sensor surface and could provide extended use. The mean peak area for the triplicate injections after 18 h of operation was 4,541 units. The raw data was smoothed using a Savitsky-Golay algorithm (40 pt window and 2nd degree polynomial fit).

At the conclusion of the field demonstration the system was retrieved from the marine environment and then subjected to direct injections of TNT in triplicate (100 ppb; prepared in seawater) (Figure 7) to determine if the TNT microfluidic immunosensor remained viable for continued use. A positive response was observed for each TNT injection post 18 h deployment suggesting continued operation in natural seawater was possible (logistics and field demonstration time constraints limited deployment time to 18 h).

These field results augment prior work reported by Green *et al.* [19] and Adams *et al.* [21] where the use of antibodies specific for TNT on two different sensor platforms (i.e., microporous beads, microchannel device) maintained functionality and viability in natural seawater. This demonstration highlights the numerous advantages of the immunosensor compared to other field deployed TNT sensing platforms: (1) higher sensitivity (parts-per-trillion to ppb levels) and (2) longevity (>18 h of continuous operation) which far exceeds previously published reports to our knowledge [21,22], (3) increased flow rates (up to 9 mL/min) resulting in improved response times (12 s) which compares favorably to many electrochemical detection methods, (4) requires no sample preconcentration which as reported by Trammell and colleagues was necessary to improve sensor sensitivity for trace level detection [23] and most importantly (5) field-durability.

## 4. Conclusions

Described here are the results of a field demonstration showing specificity and longevity of a microfluidic immunosensing device specifically tailored for trace level detection of TNT in the natural marine environment. We were successfully able to detect trace levels of TNT in natural seawater over an extended time period (>18 h; overnight operation) in a dynamic marine environment (varying tidal and current conditions) with little to no loss in sensor functionality. Detectable levels of TNT in seawater were estimated as low as 1.0 ppb based on observed fluorescence signal responses and prior calibration curves. Data telemetry using wireless communication to secure internet sites also provided the potential for remote access and data analysis. The microfluidic immunosensor showed no adverse effects to naturally occurring environmental compounds and biomass, displayed no evidence of biofouling, required no preconcentration of samples and used no organic solvents for operation. Further, this field demonstration effectively showed how the use of biomolecular components in a displacement based immunoassay can provide an alternative approach for real time detection of TNT at trace levels. Not only does it demonstrate the flexibility afforded by integrating immunomodified microfluidic technologies, but it also shows great potential for expansion to other targets of interest (i.e., toxins or TICs) in applications where long-term monitoring or sentinel detection capabilities would be beneficial.
